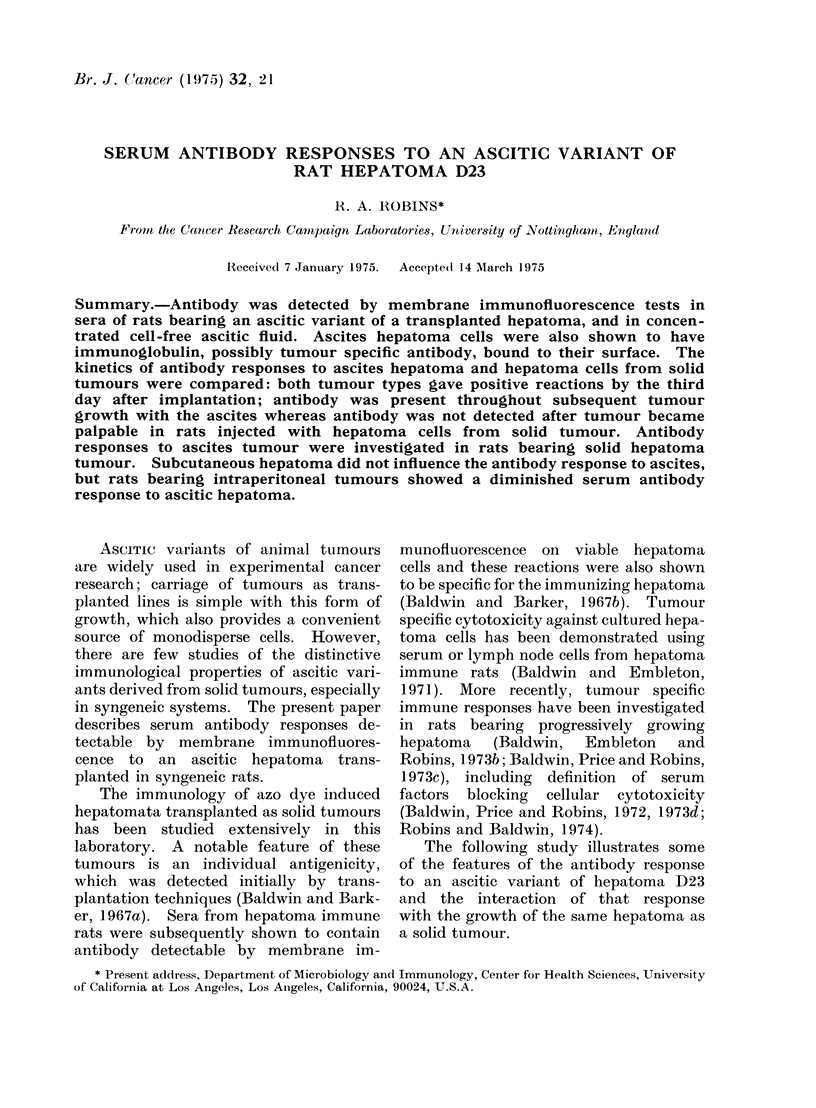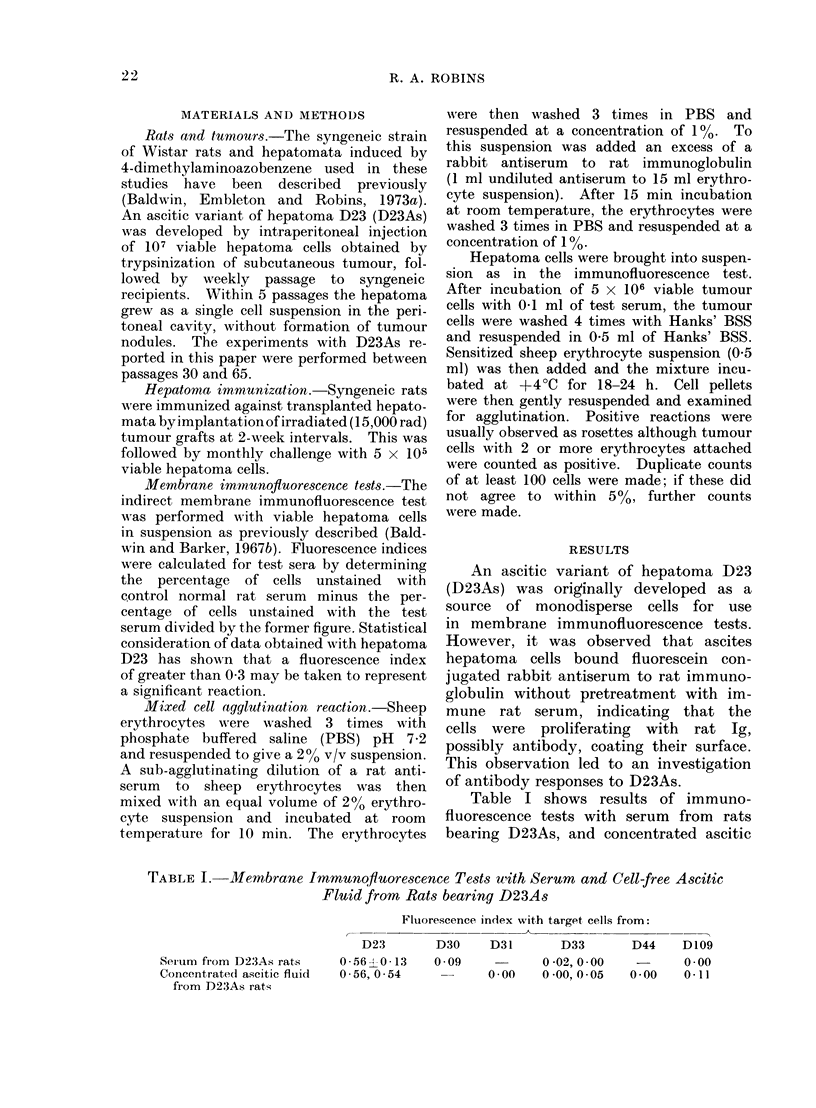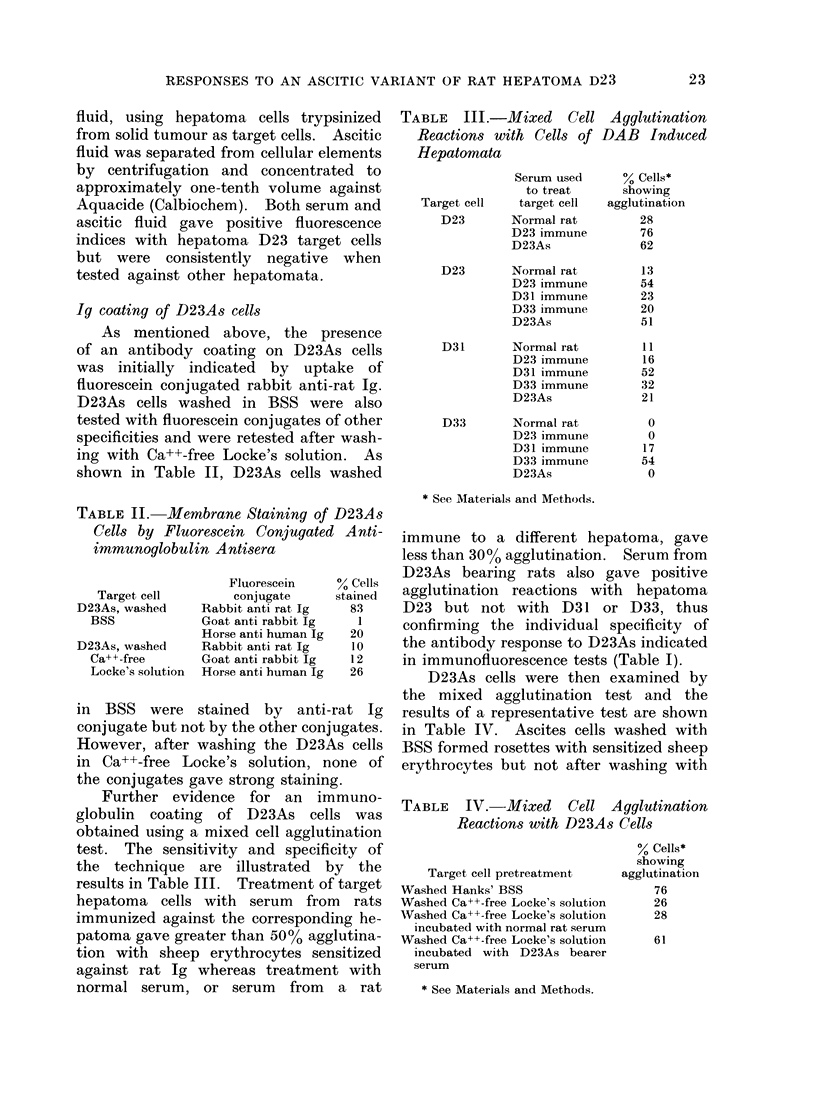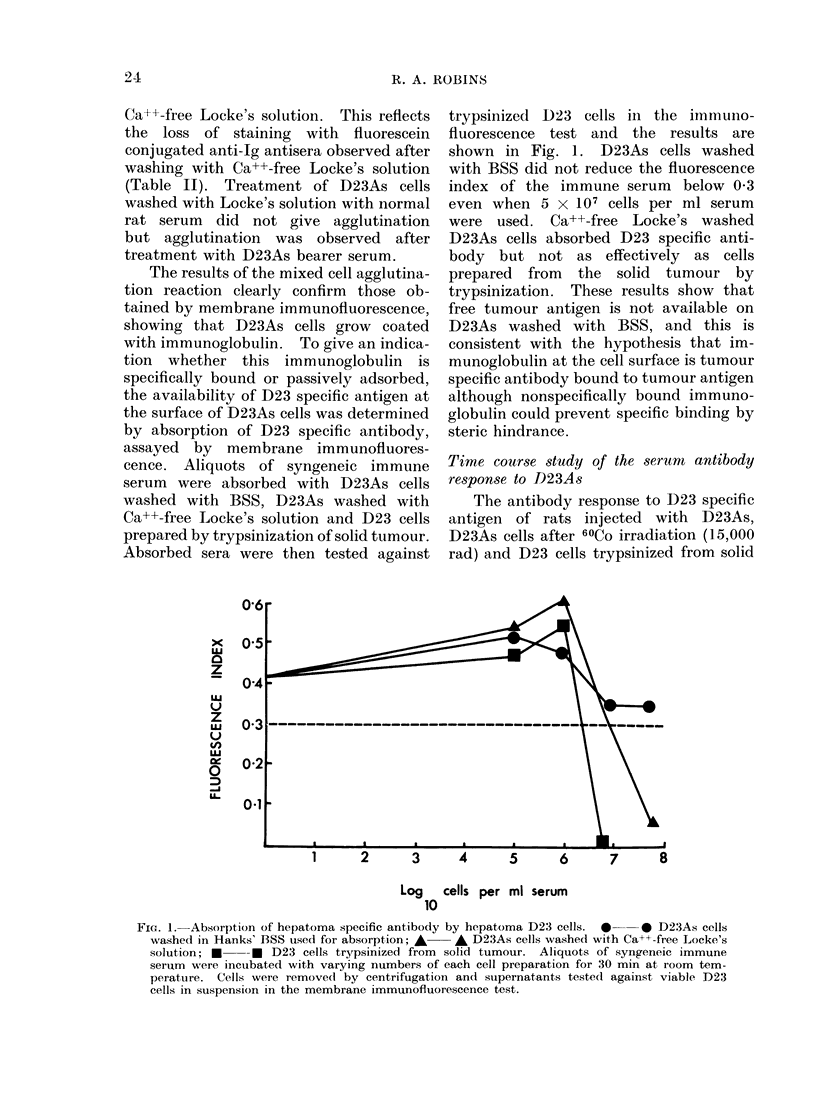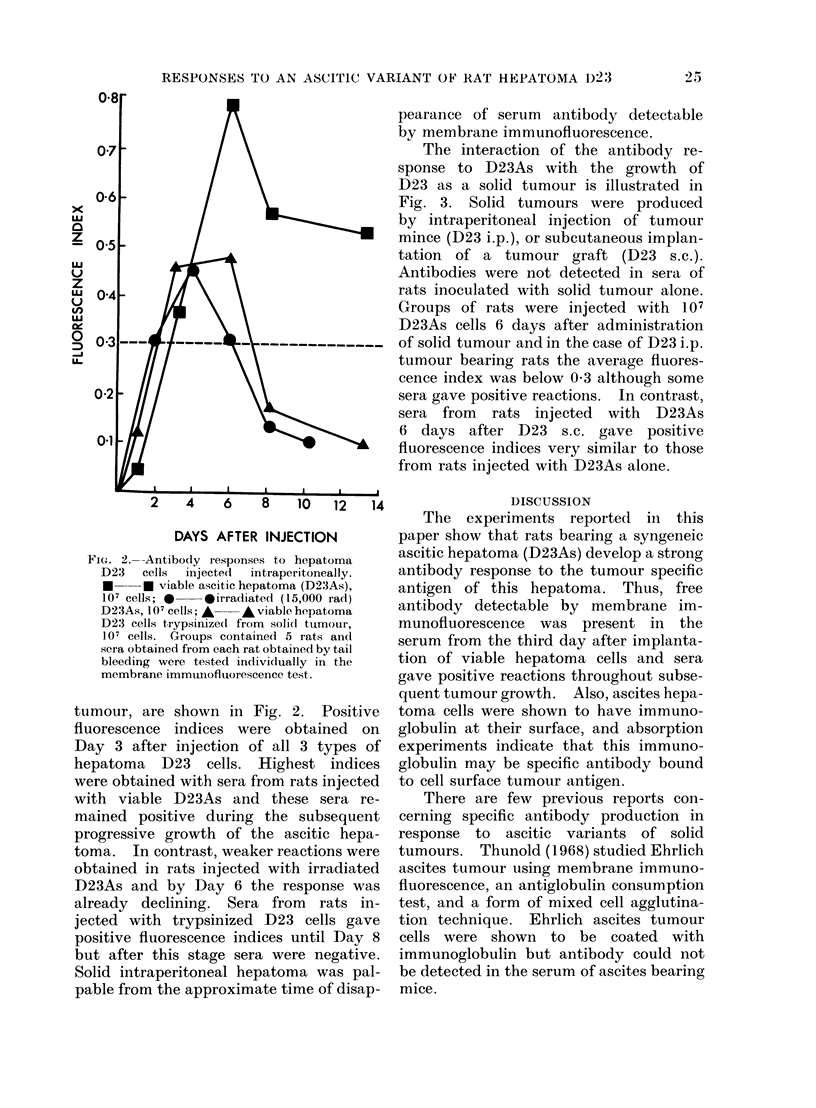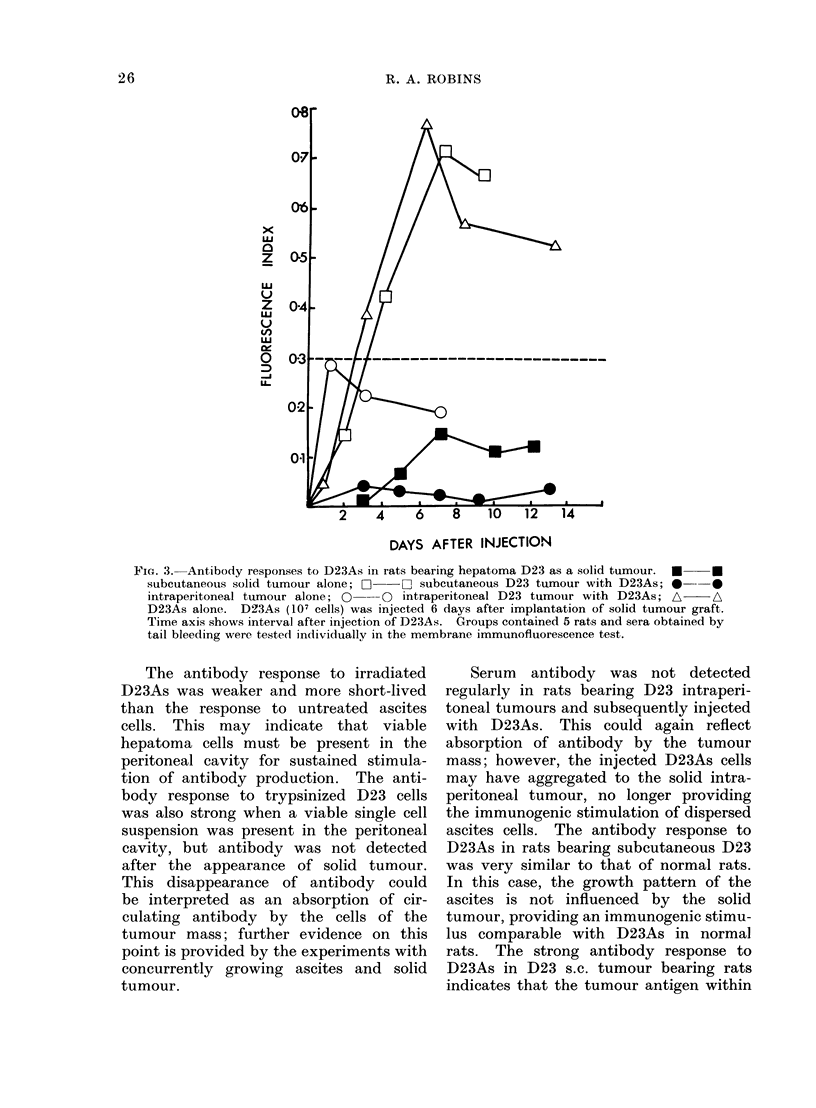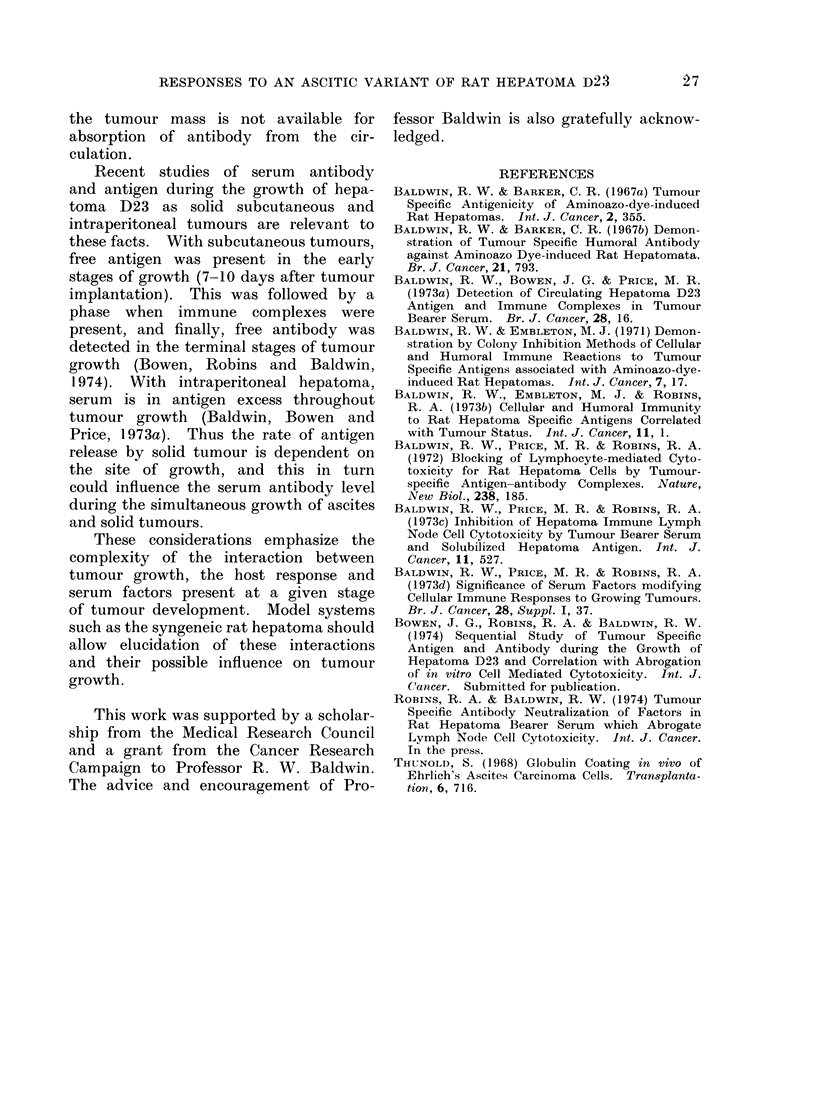# Serum antibody responses to an ascitic variant of rat hepatoma D23.

**DOI:** 10.1038/bjc.1975.129

**Published:** 1975-07

**Authors:** R. A. Robins

## Abstract

Antibody was detected by membrane immunofluorescence tests in sera of rats bearing an ascitic variant of a transplanted hepatoma, and in concentrated cell-free ascitic fluid. Ascites hepatoma cells were also shown to have immunoglobulin, possibly tumour specific antibody, bound to their surface. The kinetics of antibody responses to ascites hepatoma and hepatoma cells from solid tumours were compared: both tumour types gave positive reactions by the third day after implantation; antibody was present throughout subsequent tumour growth with the ascites whereas antibody was not detected after tumour became palpable in rats injected with hepatoma cells from solid tumour. Antibody responses to ascites tumour were investigated in rats bearing solid hepatoma tumour. Subcutaneous hepatoma did not influence the antibody response to ascites, but rats bearing intraperitoneal tumours showed a diminished serum antibody response to ascitic hepatoma.


*Br. J. Cancer* (1975) **32**, 21

### SERUM ANTIBODY RESPONSES TO AN ASCITIC VARIANT OF RAT HEPATOMA D23

R. A. ROBINS*

*From the Cancer Research Campaign Laboratories, University of Nottingham, England*

Received 7 January 1975. Accepted 14 March 1975

**Summary.—Antibody was detected by membrane immunofluorescence tests in sera of rats bearing an ascitic variant of a transplanted hepatoma, and in concentrated cell-free ascitic fluid. Ascites hepatoma cells were also shown to have immunoglobulin, possibly tumour specific antibody, bound to their surface. The kinetics of antibody responses to ascites hepatoma and hepatoma cells from solid tumours were compared: both tumour types gave positive reactions by the third day after implantation; antibody was present throughout subsequent tumour growth with the ascites whereas antibody was not detected after tumour became palpable in rats injected with hepatoma cells from solid tumour. Antibody responses to ascites tumour were investigated in rats bearing solid hepatoma tumour. Subcutaneous hepatoma did not influence the antibody response to ascites, but rats bearing intraperitoneal tumours showed a diminished serum antibody response to ascitic hepatoma.**

ASCITIC variants of animal tumours are widely used in experimental cancer research; carriage of tumours as transplanted lines is simple with this form of growth, which also provides a convenient source of monodisperse cells. However, there are few studies of the distinctive immunological properties of ascitic variants derived from solid tumours, especially in syngeneic systems. The present paper describes serum antibody responses detectable by membrane immunofluorescence to an ascitic hepatoma transplanted in syngeneic rats.

The immunology of azo dye induced hepatomata transplanted as solid tumours has been studied extensively in this laboratory. A notable feature of these tumours is an individual antigenicity, which was detected initially by transplantation techniques (Baldwin and Barker, 1967*a*). Sera from hepatoma immune rats were subsequently shown to contain antibody detectable by membrane immunofluorescence on viable hepatoma cells and these reactions were also shown to be specific for the immunizing hepatoma (Baldwin and Barker, 1967*b*). Tumour specific cytotoxicity against cultured hepatoma cells has been demonstrated using serum or lymph node cells from hepatoma immune rats (Baldwin and Embleton, 1971). More recently, tumour specific immune responses have been investigated in rats bearing progressively growing hepatoma (Baldwin, Embleton and Robins, 1973*b*; Baldwin, Price and Robins, 1973*c*), including definition of serum factors blocking cellular cytotoxicity (Baldwin, Price and Robins, 1972, 1973*d*; Robins and Baldwin, 1974).

The following study illustrates some of the features of the antibody response to an ascitic variant of hepatoma D23 and the interaction of that response with the growth of the same hepatoma as a solid tumour.

* Present address, Department of Microbiology and Immunology, Center for Health Sciences, University of California at Los Angeles, Los Angeles, California, 90024, U.S.A.

### MATERIALS AND METHODS

*Rats and tumours.*—The syngeneic strain of Wistar rats and hepatomata induced by 4-dimethylaminoazobenzene used in these studies have been described previously (Baldwin, Embleton and Robins, 1973*a*). An ascitic variant of hepatoma D23 (D23As) was developed by intraperitoneal injection of $10^7$ viable hepatoma cells obtained by trypsinization of subcutaneous tumour, followed by weekly passage to syngeneic recipients. Within 5 passages the hepatoma grew as a single cell suspension in the peritoneal cavity, without formation of tumour nodules. The experiments with D23As reported in this paper were performed between passages 30 and 65.

*Hepatoma immunization.*—Syngeneic rats were immunized against transplanted hepatomata by implantation of irradiated (15,000 rad) tumour grafts at 2-week intervals. This was followed by monthly challenge with $5 \times 10^5$ viable hepatoma cells.

*Membrane immunofluorescence tests.*—The indirect membrane immunofluorescence test was performed with viable hepatoma cells in suspension as previously described (Baldwin and Barker, 1967*b*). Fluorescence indices were calculated for test sera by determining the percentage of cells unstained with control normal rat serum minus the percentage of cells unstained with the test serum divided by the former figure. Statistical consideration of data obtained with hepatoma D23 has shown that a fluorescence index of greater than 0·3 may be taken to represent a significant reaction.

*Mixed cell agglutination reaction.*—Sheep erythrocytes were washed 3 times with phosphate buffered saline (PBS) pH 7·2 and resuspended to give a 2% v/v suspension. A sub-agglutinating dilution of a rat antiserum to sheep erythrocytes was then mixed with an equal volume of 2% erythrocyte suspension and incubated at room temperature for 10 min. The erythrocytes were then washed 3 times in PBS and resuspended at a concentration of 1%. To this suspension was added an excess of a rabbit antiserum to rat immunoglobulin (1 ml undiluted antiserum to 15 ml erythrocyte suspension). After 15 min incubation at room temperature, the erythrocytes were washed 3 times in PBS and resuspended at a concentration of 1%.

Hepatoma cells were brought into suspension as in the immunofluorescence test. After incubation of $5 \times 10^6$ viable tumour cells with 0·1 ml of test serum, the tumour cells were washed 4 times with Hanks' BSS and resuspended in 0·5 ml of Hanks' BSS. Sensitized sheep erythrocyte suspension (0·5 ml) was then added and the mixture incubated at +4°C for 18–24 h. Cell pellets were then gently resuspended and examined for agglutination. Positive reactions were usually observed as rosettes although tumour cells with 2 or more erythrocytes attached were counted as positive. Duplicate counts of at least 100 cells were made; if these did not agree to within 5%, further counts were made.

### RESULTS

An ascitic variant of hepatoma D23 (D23As) was originally developed as a source of monodisperse cells for use in membrane immunofluorescence tests. However, it was observed that ascites hepatoma cells bound fluorescein conjugated rabbit antiserum to rat immunoglobulin without pretreatment with immune rat serum, indicating that the cells were proliferating with rat Ig, possibly antibody, coating their surface. This observation led to an investigation of antibody responses to D23As.

Table I shows results of immunofluorescence tests with serum from rats bearing D23As, and concentrated ascitic

TABLE I.—*Membrane Immunofluorescence Tests with Serum and Cell-free Ascitic Fluid from Rats bearing D23As*

| | Fluorescence index with target cells from: | | | | | |
|---|---|---|---|---|---|---|
| | D23 | D30 | D31 | D33 | D44 | D109 |
| Serum from D23As rats | 0·56±0·13 | 0·09 | — | 0·02, 0·00 | — | 0·00 |
| Concentrated ascitic fluid from D23As rats | 0·56, 0·54 | — | 0·00 | 0·00, 0·05 | 0·00 | 0·11 |

fluid, using hepatoma cells trypsinized from solid tumour as target cells. Ascitic fluid was separated from cellular elements by centrifugation and concentrated to approximately one-tenth volume against Aquacide (Calbiochem). Both serum and ascitic fluid gave positive fluorescence indices with hepatoma D23 target cells but were consistently negative when tested against other hepatomata.

*Ig coating of D23As cells*

As mentioned above, the presence of an antibody coating on D23As cells was initially indicated by uptake of fluorescein conjugated rabbit anti-rat Ig. D23As cells washed in BSS were also tested with fluorescein conjugates of other specificities and were retested after washing with $Ca^{++}$-free Locke's solution. As shown in Table II, D23As cells washed in BSS were stained by anti-rat Ig conjugate but not by the other conjugates. However, after washing the D23As cells in $Ca^{++}$-free Locke's solution, none of the conjugates gave strong staining.

TABLE II.—*Membrane Staining of D23As Cells by Fluorescein Conjugated Anti-immunoglobulin Antisera*

| Target cell | Fluorescein conjugate | % Cells stained |
|---|---|---|
| D23As, washed BSS | Rabbit anti rat Ig | 83 |
| | Goat anti rabbit Ig | 1 |
| | Horse anti human Ig | 20 |
| D23As, washed $Ca^{++}$-free Locke's solution | Rabbit anti rat Ig | 10 |
| | Goat anti rabbit Ig | 12 |
| | Horse anti human Ig | 26 |

Further evidence for an immunoglobulin coating of D23As cells was obtained using a mixed cell agglutination test. The sensitivity and specificity of the technique are illustrated by the results in Table III. Treatment of target hepatoma cells with serum from rats immunized against the corresponding hepatoma gave greater than 50% agglutination with sheep erythrocytes sensitized against rat Ig whereas treatment with normal serum, or serum from a rat immune to a different hepatoma, gave less than 30% agglutination. Serum from D23As bearing rats also gave positive agglutination reactions with hepatoma D23 but not with D31 or D33, thus confirming the individual specificity of the antibody response to D23As indicated in immunofluorescence tests (Table I).

TABLE III.—*Mixed Cell Agglutination Reactions with Cells of DAB Induced Hepatomata*

| Target cell | Serum used to treat target cell | % Cells* showing agglutination |
|---|---|---|
| D23 | Normal rat | 28 |
| | D23 immune | 76 |
| | D23As | 62 |
| D23 | Normal rat | 13 |
| | D23 immune | 54 |
| | D31 immune | 23 |
| | D33 immune | 20 |
| | D23As | 51 |
| D31 | Normal rat | 11 |
| | D23 immune | 16 |
| | D31 immune | 52 |
| | D33 immune | 32 |
| | D23As | 21 |
| D33 | Normal rat | 0 |
| | D23 immune | 0 |
| | D31 immune | 17 |
| | D33 immune | 54 |
| | D23As | 0 |

* See Materials and Methods.

D23As cells were then examined by the mixed agglutination test and the results of a representative test are shown in Table IV. Ascites cells washed with BSS formed rosettes with sensitized sheep erythrocytes but not after washing with

TABLE IV.—*Mixed Cell Agglutination Reactions with D23As Cells*

| Target cell pretreatment | % Cells* showing agglutination |
|---|---|
| Washed Hanks' BSS | 76 |
| Washed $Ca^{++}$-free Locke's solution | 26 |
| Washed $Ca^{++}$-free Locke's solution incubated with normal rat serum | 28 |
| Washed $Ca^{++}$-free Locke's solution incubated with D23As bearer serum | 61 |

* See Materials and Methods.

$Ca^{++}$-free Locke's solution. This reflects the loss of staining with fluorescein conjugated anti-Ig antisera observed after washing with $Ca^{++}$-free Locke's solution (Table II). Treatment of D23As cells washed with Locke's solution with normal rat serum did not give agglutination but agglutination was observed after treatment with D23As bearer serum.

The results of the mixed cell agglutination reaction clearly confirm those obtained by membrane immunofluorescence, showing that D23As cells grow coated with immunoglobulin. To give an indication whether this immunoglobulin is specifically bound or passively adsorbed, the availability of D23 specific antigen at the surface of D23As cells was determined by absorption of D23 specific antibody, assayed by membrane immunofluorescence. Aliquots of syngeneic immune serum were absorbed with D23As cells washed with BSS, D23As washed with $Ca^{++}$-free Locke's solution and D23 cells prepared by trypsinization of solid tumour. Absorbed sera were then tested against trypsinized D23 cells in the immunofluorescence test and the results are shown in Fig. 1. D23As cells washed with BSS did not reduce the fluorescence index of the immune serum below 0·3 even when $5 \times 10^7$ cells per ml serum were used. $Ca^{++}$-free Locke's washed D23As cells absorbed D23 specific antibody but not as effectively as cells prepared from the solid tumour by trypsinization. These results show that free tumour antigen is not available on D23As washed with BSS, and this is consistent with the hypothesis that immunoglobulin at the cell surface is tumour specific antibody bound to tumour antigen although nonspecifically bound immunoglobulin could prevent specific binding by steric hindrance.

*Time course study of the serum antibody response to D23As*

The antibody response to D23 specific antigen of rats injected with D23As, D23As cells after $^{60}Co$ irradiation (15,000 rad) and D23 cells trypsinized from solid

FIG. 1.—Absorption of hepatoma specific antibody by hepatoma D23 cells. ●——● D23As cells washed in Hanks' BSS used for absorption; ▲——▲ D23As cells washed with $Ca^{++}$-free Locke's solution; ■——■ D23 cells trypsinized from solid tumour. Aliquots of syngeneic immune serum were incubated with varying numbers of each cell preparation for 30 min at room temperature. Cells were removed by centrifugation and supernatants tested against viable D23 cells in suspension in the membrane immunofluorescence test.

FIG. 2.—Antibody responses to hepatoma D23 cells injected intraperitoneally. ■——■ viable ascitic hepatoma (D23As), $10^7$ cells; ●——●irradiated (15,000 rad) D23As, $10^7$ cells; ▲——▲viable hepatoma D23 cells trypsinized from solid tumour, $10^7$ cells. Groups contained 5 rats and sera obtained from each rat obtained by tail bleeding were tested individually in the membrane immunofluorescence test.

tumour, are shown in Fig. 2. Positive fluorescence indices were obtained on Day 3 after injection of all 3 types of hepatoma D23 cells. Highest indices were obtained with sera from rats injected with viable D23As and these sera remained positive during the subsequent progressive growth of the ascitic hepatoma. In contrast, weaker reactions were obtained in rats injected with irradiated D23As and by Day 6 the response was already declining. Sera from rats injected with trypsinized D23 cells gave positive fluorescence indices until Day 8 but after this stage sera were negative. Solid intraperitoneal hepatoma was palpable from the approximate time of disappearance of serum antibody detectable by membrane immunofluorescence.

The interaction of the antibody response to D23As with the growth of D23 as a solid tumour is illustrated in Fig. 3. Solid tumours were produced by intraperitoneal injection of tumour mince (D23 i.p.), or subcutaneous implantation of a tumour graft (D23 s.c.). Antibodies were not detected in sera of rats inoculated with solid tumour alone. Groups of rats were injected with $10^7$ D23As cells 6 days after administration of solid tumour and in the case of D23 i.p. tumour bearing rats the average fluorescence index was below 0·3 although some sera gave positive reactions. In contrast, sera from rats injected with D23As 6 days after D23 s.c. gave positive fluorescence indices very similar to those from rats injected with D23As alone.

### DISCUSSION

The experiments reported in this paper show that rats bearing a syngeneic ascitic hepatoma (D23As) develop a strong antibody response to the tumour specific antigen of this hepatoma. Thus, free antibody detectable by membrane immunofluorescence was present in the serum from the third day after implantation of viable hepatoma cells and sera gave positive reactions throughout subsequent tumour growth. Also, ascites hepatoma cells were shown to have immunoglobulin at their surface, and absorption experiments indicate that this immunoglobulin may be specific antibody bound to cell surface tumour antigen.

There are few previous reports concerning specific antibody production in response to ascitic variants of solid tumours. Thunold (1968) studied Ehrlich ascites tumour using membrane immunofluorescence, an antiglobulin consumption test, and a form of mixed cell agglutination technique. Ehrlich ascites tumour cells were shown to be coated with immunoglobulin but antibody could not be detected in the serum of ascites bearing mice.

FIG. 3.—Antibody responses to D23As in rats bearing hepatoma D23 as a solid tumour. ■——■ subcutaneous solid tumour alone; □——□ subcutaneous D23 tumour with D23As; ●——● intraperitoneal tumour alone; ○——○ intraperitoneal D23 tumour with D23As; △——△ D23As alone. D23As ($10^7$ cells) was injected 6 days after implantation of solid tumour graft. Time axis shows interval after injection of D23As. Groups contained 5 rats and sera obtained by tail bleeding were tested individually in the membrane immunofluorescence test.

The antibody response to irradiated D23As was weaker and more short-lived than the response to untreated ascites cells. This may indicate that viable hepatoma cells must be present in the peritoneal cavity for sustained stimulation of antibody production. The antibody response to trypsinized D23 cells was also strong when a viable single cell suspension was present in the peritoneal cavity, but antibody was not detected after the appearance of solid tumour. This disappearance of antibody could be interpreted as an absorption of circulating antibody by the cells of the tumour mass; further evidence on this point is provided by the experiments with concurrently growing ascites and solid tumour.

Serum antibody was not detected regularly in rats bearing D23 intraperitoneal tumours and subsequently injected with D23As. This could again reflect absorption of antibody by the tumour mass; however, the injected D23As cells may have aggregated to the solid intraperitoneal tumour, no longer providing the immunogenic stimulation of dispersed ascites cells. The antibody response to D23As in rats bearing subcutaneous D23 was very similar to that of normal rats. In this case, the growth pattern of the ascites is not influenced by the solid tumour, providing an immunogenic stimulus comparable with D23As in normal rats. The strong antibody response to D23As in D23 s.c. tumour bearing rats indicates that the tumour antigen within the tumour mass is not available for absorption of antibody from the circulation.

Recent studies of serum antibody and antigen during the growth of hepatoma D23 as solid subcutaneous and intraperitoneal tumours are relevant to these facts. With subcutaneous tumours, free antigen was present in the early stages of growth (7–10 days after tumour implantation). This was followed by a phase when immune complexes were present, and finally, free antibody was detected in the terminal stages of tumour growth (Bowen, Robins and Baldwin, 1974). With intraperitoneal hepatoma, serum is in antigen excess throughout tumour growth (Baldwin, Bowen and Price, 1973*a*). Thus the rate of antigen release by solid tumour is dependent on the site of growth, and this in turn could influence the serum antibody level during the simultaneous growth of ascites and solid tumours.

These considerations emphasize the complexity of the interaction between tumour growth, the host response and serum factors present at a given stage of tumour development. Model systems such as the syngeneic rat hepatoma should allow elucidation of these interactions and their possible influence on tumour growth.

This work was supported by a scholarship from the Medical Research Council and a grant from the Cancer Research Campaign to Professor R. W. Baldwin. The advice and encouragement of Professor Baldwin is also gratefully acknowledged.

REFERENCES

Baldwin, R. W. & Barker, C. R. (1967*a*) Tumour Specific Antigenicity of Aminoazo-dye-induced Rat Hepatomas. *Int. J. Cancer*, **2**, 355.

Baldwin, R. W. & Barker, C. R. (1967*b*) Demonstration of Tumour Specific Humoral Antibody against Aminoazo Dye-induced Rat Hepatomata. *Br. J. Cancer*, **21**, 793.

Baldwin, R. W., Bowen, J. G. & Price, M. R. (1973*a*) Detection of Circulating Hepatoma D23 Antigen and Immune Complexes in Tumour Bearer Serum. *Br. J. Cancer*, **28**, 16.

Baldwin, R. W. & Embleton, M. J. (1971) Demonstration by Colony Inhibition Methods of Cellular and Humoral Immune Reactions to Tumour Specific Antigens associated with Aminoazo-dye-induced Rat Hepatomas. *Int. J. Cancer*, **7**, 17.

Baldwin, R. W., Embleton, M. J. & Robins, R. A. (1973*b*) Cellular and Humoral Immunity to Rat Hepatoma Specific Antigens Correlated with Tumour Status. *Int. J. Cancer*, **11**, 1.

Baldwin, R. W., Price, M. R. & Robins, R. A. (1972) Blocking of Lymphocyte-mediated Cytotoxicity for Rat Hepatoma Cells by Tumour-specific Antigen–antibody Complexes. *Nature, New Biol.*, **238**, 185.

Baldwin, R. W., Price, M. R. & Robins, R. A. (1973*c*) Inhibition of Hepatoma Immune Lymph Node Cell Cytotoxicity by Tumour Bearer Serum and Solubilized Hepatoma Antigen. *Int. J. Cancer*, **11**, 527.

Baldwin, R. W., Price, M. R. & Robins, R. A. (1973*d*) Significance of Serum Factors modifying Cellular Immune Responses to Growing Tumours. *Br. J. Cancer*, **28**, *Suppl.* I, 37.

Bowen, J. G., Robins, R. A. & Baldwin, R. W. (1974) Sequential Study of Tumour Specific Antigen and Antibody during the Growth of Hepatoma D23 and Correlation with Abrogation of *in vitro* Cell Mediated Cytotoxicity. *Int. J. Cancer*. Submitted for publication.

Robins, R. A. & Baldwin, R. W. (1974) Tumour Specific Antibody Neutralization of Factors in Rat Hepatoma Bearer Serum which Abrogate Lymph Node Cell Cytotoxicity. *Int. J. Cancer*. In the press.

Thunold, S. (1968) Globulin Coating *in vivo* of Ehrlich's Ascites Carcinoma Cells. *Transplantation*, **6**, 716.